# Triple-0: Zero-shot denoising and dereverberation on an end-to-end frozen anechoic speech separation network

**DOI:** 10.1371/journal.pone.0301692

**Published:** 2024-07-16

**Authors:** Sania Gul, Muhammad Salman Khan, Ata Ur-Rehman

**Affiliations:** 1 Department of Electrical Engineering, University of Engineering and Technology, Peshawar, Pakistan; 2 Intelligent Information Processing Lab, National Center of Artificial Intelligence, University of Engineering and Technology, Peshawar, Pakistan; 3 Department of Electrical Engineering, College of Engineering, Qatar University, Doha, Qatar; 4 Department of Electrical Engineering, MCS, NUST, Islamabad, Pakistan; 5 Department of Business and Computing, Ravensbourne University London, London, United Kingdom; Guangdong University of Petrochemical Technology, CHINA

## Abstract

Speech enhancement is crucial both for human and machine listening applications. Over the last decade, the use of deep learning for speech enhancement has resulted in tremendous improvement over the classical signal processing and machine learning methods. However, training a deep neural network is not only time-consuming; it also requires extensive computational resources and a large training dataset. Transfer learning, i.e. using a pretrained network for a new task, comes to the rescue by reducing the amount of training time, computational resources, and the required dataset, but the network still needs to be fine-tuned for the new task. This paper presents a novel method of speech denoising and dereverberation (SD&D) on an end-to-end frozen binaural anechoic speech separation network. The frozen network requires neither any architectural change nor any fine-tuning for the new task, as is usually required for transfer learning. The interaural cues of a source placed inside noisy and echoic surroundings are given as input to this pretrained network to extract the target speech from noise and reverberation. Although the pretrained model used in this paper has never seen noisy reverberant conditions during its training, it performs satisfactorily for zero-shot testing (ZST) under these conditions. It is because the pretrained model used here has been trained on the direct-path interaural cues of an active source and so it can recognize them even in the presence of echoes and noise. ZST on the same dataset on which the pretrained network was trained (homo-corpus) for the unseen class of interference, has shown considerable improvement over the weighted prediction error (WPE) algorithm in terms of four objective speech quality and intelligibility metrics. Also, the proposed model offers similar performance provided by a deep learning SD&D algorithm for this dataset under varying conditions of noise and reverberations. Similarly, ZST on a different dataset has provided an improvement in intelligibility and almost equivalent quality as provided by the WPE algorithm.

## Introduction

**Fig 1:**
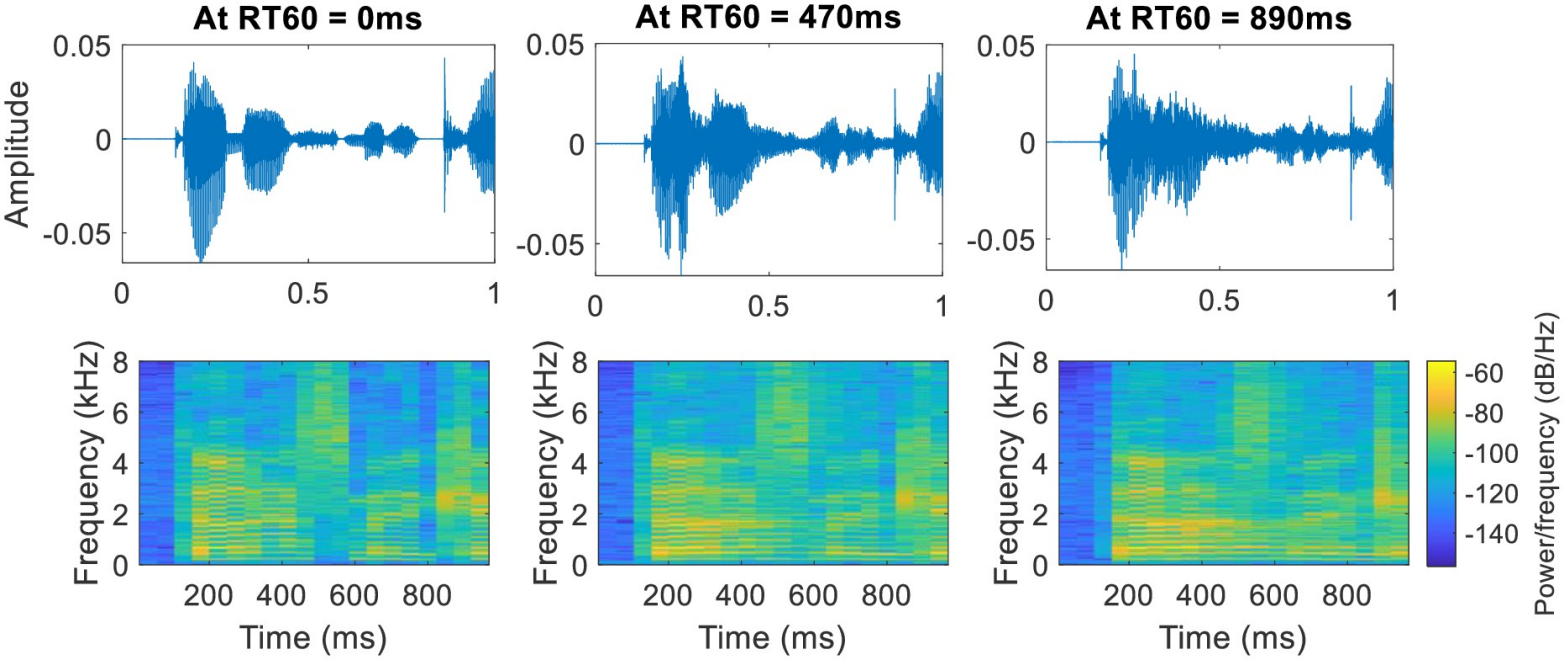
Depiction of smearing effect in time-domain (top) and time-frequency domain signal (bottom) with changing *RT*_*60*_. The smearing effect increases with *RT*_*60*_.

While traveling through the air, the two main factors contaminating the sound before it reaches the listener are: 1) noise, and 2) reverberations ([1, 2]). Noise usually refers to the sounds produced by other sources. Noise results in deteriorating the quality of the received sound, and reducing its intelligibility, in case if the sound is a speech signal [3]. Reverberation is a noise produced by the source itself [4]. Reverberation is a natural phenomenon present almost everywhere around us e.g. in concert halls, offices, stairways, city streets, and even in the woods [5]. For machine listening, the presence of noise and reverberation results in difficulty in automatic speech recognition, speaker verification, source separation, and source localization ([6, 7]). For biomedical, bioacoustics, industrial, and environmental sounds, the presence of reverberation and noise results in reducing the accuracy of separation and classification of different components in an audio mixture [8]. However, music without a moderate amount of reverberation sounds lifeless and dry. But, too much reverberation results in sounding a fine musical performance unintelligible and muddy [5]. In the case of speech, reverberations result in loss of intelligibility for people who are hard of hearing and non-native listeners in noiseless enclosures. In noisy enclosures, reverberations reduce the intelligibility even for people with normal listening [9]. Both noise and reverberation play havoc when the generated speech is already less intelligible (e.g. spoken by a person suffering from medical conditions causing speech dysarthria [10]). Subjective assessment of noisy Ukrainian speech shows that for 0.6–0.8 m distance between the speaker and listener, reverberation has practically no effect on intelligibility [11]. However, the effect becomes prominent after this distance. The reduction in intelligibility by reverberation is caused by the smearing effect that rises with the reverberation time (*RT*_*60*_) as depicted in Fig 1.

**Fig 2:**
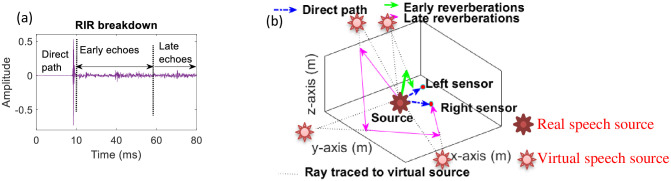
(a) RIR decomposition in three components i.e. 1) direct-path, 2) early echoes and 3) late reflections, marked on RIR signal (b) Creation of virtual sources by reverberations. All sources (real and virtual) generate their distinguishing interaural cues.

Noise is an additive distortion, while the reverberations are modelled as convolutive distortions [12]. The signal emitted from source gets distorted while travelling towards receiver, due to its convolution with the room impulse response (RIR). RIR is the transfer function between the source and the microphone that characterizes the path between the speaker and the listener [13]. A sound emitted from a source placed inside an enclosure reaches the listener both by the direct-path (corresponding to the line-of-sight propagation) and by the reflections off the surrounding objects and walls. The first waves that reach the listener from a source are the direct-path waves and those reaching within 50ms after the direct-path signals are called as the early echoes (reverberations). Early reverberations stem from a few disjoint specular reflections from large flat surfaces such as walls of the room. The waves reaching after 50ms of the direct wave are termed as the late reverberations and comprise dense reflections produced by scattering effects caused by sound’s interaction with rough surfaces and small objects [14]. The RIR decomposition in three components i.e. direct-path signals, early echoes, and late reverberations is shown in Fig 2(a), where the RIR is plotted as a time-domain signal and the y-axis represents the amplitude of the signal at the receiver (microphone), when the source emits an impulse [14].

Although the early reflections increase the signal-to-noise ratio (SNR) up to 6dB and sometimes even by 9dB, their effect on intelligibility is somewhat complex [15]. The early reflections increase the intelligibility for a listener near the walls due to the increased energy of signal by the combined action of direct sound and the early reflections. However, the intelligibility reduces to its minimal value in the middle of the room not only because of reduction in quality caused by the late reverberations but also due to early reverberations, which cause an uneven frequency response of the room [15].

Early reverberations are specular (i.e. they can be considered as distinct sources on their own), while the late echoes are modeled as a diffuse noise source [16]. Both early and late reflections are characterized by their delayed arrival and reduced strength as compared to the direct-path waves [12]. Furthermore, the specular reflections are characterized by their distinguishing interaural cues. These cues are also called as spatial (as they are indicative of the source spatial position) or binaural cues (as they exist due to the spacing between the two ears). There are two important types of interaural cues 1) the interaural level difference (ILD) and 2) the interaural time difference (ITD) used for source localization by human beings. ITD is related to the delay between the two ears. It is effective for low frequencies. ILD is the difference in intensity of sound reaching the two ears [17]. ILD is useful at high frequencies. Both ILD and ITD are zero for the source on the front plane [17]; the median-sagittal plane (the plane running from head to toe bisecting the left and right sides of the body [7]). Human beings use these cues to localize the acoustic sources in azimuth and also in the elevation when the source is off the median-sagittal plane [16]. Due to their specular nature, the early reflections can be imagined as the signals coming from several virtual sources (producing weak and delayed replicas of the original source) [18]. As these virtual sources are located at different positions in an enclosure [18], their spatial cues are different from the cues of the direct-path waves, which indicate the position of the real-source ([19, 20]), as shown in Fig 2(b). Similar to reverberation, the noise source can be directional (focused) or it is diffuse (background noise) [21]. It is shown in [22, 23] that the noise effect on hearing is much stronger than reverberation. Subjective listening tests in [15] show that the noise inside a room is a much greater danger to intelligibility than the reverberations.

The goal of speech enhancement (SE) algorithms is to remove unwanted noise and reverberations from speech to improve its quality and intelligibility [4]. Although many signal processing and machine learning techniques are still in use to combat the fatal effects of noise and reverberations, speech denoising and dereverberation (SD&D) by deep neural networks have produced better results in terms of speech quality and intelligibility (e.g. [12, 24, 25]). However, the drawbacks of deep learning methods are their long training durations, increased computational cost, and large datasets required for the training /testing of models [26]. In such cases, the pretrained networks; already trained over large datasets, are very useful, as they require far fewer computational resources, data, and time than needed if the system is trained from scratch [27]. Reusing a pretrained network on a different but related problem is called transfer learning [27]. In transfer learning, the parameters of the inner layers of a pretrained network are frozen, while the output layers are customized and their parameters are fine-tuned by retraining on the dataset of the new task [28].

Another problem with deep learning models is their inability to tackle the unseen conditions encountered during testing [29]. In such cases, their performance usually degrades. The deep learning models cannot replicate the human ability to generalize and recognize objects they have never seen before [30]. As a response to these limitations, zero-shot learning has emerged in the last decade to handle unknown classes during testing [30]. Zero-data or zero-shot learning is a task of object recognition for classes for which there are no training instances [31]. Zero-shot learning is a scenario in machine learning where the classes used in the training and the testing sets are disjoint. First appeared in 2009 [32], for computer vision applications, zero-shot learning has been used for various audio applications including voice conversion (e.g. [33–35]), source separation (e.g. in [36, 37]), audio bandwidth extension [38], speech emotion recognition [39], speech-to-speech translation [40], speech recognition [41], text-to-speech conversion [42], environmental sounds classification [32], speech synthesis [43], hate speech detection [44] and so on.

In this paper, a new SD&D psychoacoustic algorithm is proposed using a binaural setup to collect the signal. From these signals, interaural cues [45] are extracted to discriminate between the direct and reverberant noisy components of speech. In reference [46], it is shown that binaural listening improves the signal-to-noise ratio (SNR) by 2dB. Our proposed model is based on a pretrained anechoic speech separation network ‘SONET’ [47]. Unlike transfer learning, where fine-tuning on the new task is required, our proposed system is capable of performing well on a frozen pretrained network. This is because of the underlying fact that the pretrained network used in our proposed system was well-trained over the direct-path interaural cues of an active source and able to recognize them even in the presence of noise and echoes. Our proposed system is named as “Triple-0”, where the first 0 represents zero architectural change in the pretrained model [47], the second for zero fine-tuning i.e. zero training data needed for the new task, and the third for the zero-shot learning.

### Related work of zero-shot learning for speech enhancement model

In speech enhancement (SE) applications, zero-shot learning is used in [48] for the selection of the most optimal SE model from the ensemble of pretrained models. In this model, different convolutional neural network (CNN)-based speech denoising (SD) models are trained with a subset of training data in the offline mode. The data in each subset is clustered based on their quality score. In the online mode, the most appropriate model for the incoming noisy speech is selected by the nearness of its quality score with the centroid value for each model. The model does not support dereverberation or cross-corpus SE tasks. The personalized SD model proposed in [49] is again an ensemble of specialist modules, each trained on a group of speakers with similar voice characteristics. The specific module for the incoming speaker is selected based on its feature matching with a particular trained module, decided by the probability calculated by the gating module. The model is tested only for noisy speech (without echoes) in the zero-shot mode. The text query-based SE model of [50] is an important step towards universal sound separation (USS) which aims to separate arbitrary sounds from real-world recordings. The system uses the ResU-Net architecture of [51] for audio separation and pretrained models [52, 53] for extracting text embeddings from the labels written in natural language. The system is trained over a large amount of speech, music, and environmental sound datasets and performs well over signal processing and deep learning audio-based methods for unseen data. However, this system is also neither trained nor tested for reverberant conditions. As in the real world the clean sources (required as references) are hardly available, the model of [54]; an unsupervised model, uses a mixture of mixtures (MoMs) as input and the original mixture itself as a reference instead of a clean source to minimize the training loss. The model uses the improved time-domain convolutional neural network (TDCNN++) architecture of [55] as its separation model and performs well on noisy reverberant data. However, the data for training and testing is created by using synthetic RIRs instead of real ones. Similarly, the speech enhancement generative adversarial network (SEGAN) [56], designed for speech denoising, failed to be effective in reducing echoes when tested for speech dereverberation [24], even after its training on the reverberant dataset. Apart from the zero-shot speech enhancement models discussed above, an SD&D algorithm using the interaural coherence (IC), ILD, and ITD cues is presented in reference [57], where a feed-forward network is trained on these cues to extract the time-frequency mask for extracting clean speech. The system is trained on anechoic noisy data but later tested for noisy reverberant conditions.

### Contribution of this paper

The main contributions of this paper are summarized below.

- To the best of our knowledge, this is the first time that this paper introduces zero-shot learning for SD&D on a pretrained binaural network, which has not been trained on noisy reverberant speech. The binaural SD&D model [57] is zero-shot tested for unseen reverberant conditions, but unlike our proposed model, that model [57] has seen the noise during its training phase. Also, [57] is designed and trained especially for SD&D, while our proposed model is reusing a speech separation model for this purpose without any need of its retraining and architectural change. Unlike the zero-shot SE models [48, 49, 51], our proposed network has been tested for reverberant conditions along with noisy ones. Also, unlike [55], our proposed system is tested for reverberant speech using real RIRs instead of synthetic ones.
- Unlike all the zero-shot models discussed above where the training is done over a large number of noisy datasets to enable these systems to differentiate the noisy content from the clean speech, no such training is done before testing our pretrained anechoic speech separation model on zero-data, saving both computational resources and training time.
- In the case of U-Net-based audio-only SE models, this is the first time that a pretrained model has been used for SD&D. In the past, pretrained U-Net-based models were used for the SE tasks of speech inpainting (e.g. in [54, 55]) and source separation (e.g. speech separation models using other modalities (e.g. video in [58, 59] and text in [51]) or audio-only models (e.g. in [47], where only a part of the pretrained model that is used here was utilized) but was never tried for SD&D.

The rest of the paper is organized as follows. The next section provides an overview of our proposed system including the pretrained model SONET. After that, the dataset, experimental settings, evaluation metrics, and the baseline algorithms are described. The comparison of different speech enhancement models is done in the second last section, after which the paper is concluded.

## Method

Before describing details of our proposed SD&D algorithm ‘Triple-0’, an overview of the pretrained model SONET (used in Triple-0) is given.

### Pretrained network SONET

In this section, we briefly discuss the pretrained speech separation neural network ‘SONET’ [47] used in our proposed SD&D algorithm Triple-0. SONET [47] is a binaural speech separation system designed for separating a speech mixture created by two spatially separated sources, placed in an anechoic condition, based on their interaural cues. It is a U-Net-based speech separation model. Although U-Nets were initially developed for medical image segmentation [60], since then, they have been widely used for audio enhancement applications in music, industrial sounds, biomedical signal, environmental sounds, and bio-acoustic signals [8]. For speech, apart from the vanilla U-Net model which requires the conversion of raw-audio to an image (usually in the form of spectrogram, which is basically an audio visualization showing the signal’s spectral contents and their change over time [61]), several architectures of U-Net e.g. wave-u-net and dilated wave-u-net and 1D U-Nets can accept the audio data directly. However it is found that time-frequency methods give better results than the algorithms accepting the raw signals directly [62].

**Fig 3:**
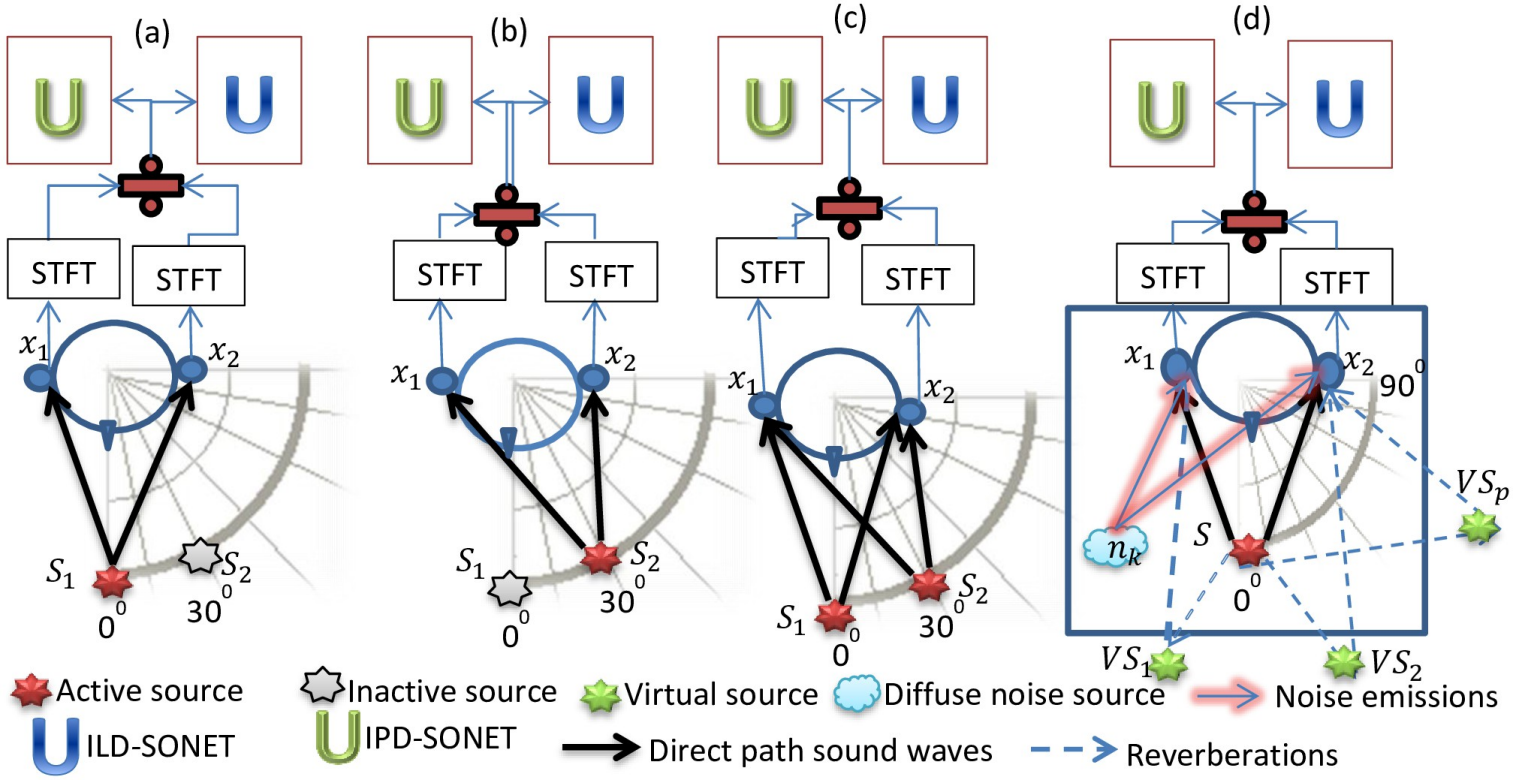
Training ((a) and (b)) and testing ((c)) phases of SONET in anechoic chamber for speech separation. Zero-shot testing of pretrained SONET in noisy echoic chamber (d).

Although SONET is a spatial cue-based system designed for target speech separation from an audio mixture, it was exposed to the interaural cues of individual sources, rather than those of speech mixture, during its training phase. The first class of its training dataset consists of interaural spectrograms of the target and the second class belongs to the interferer. The training spectrograms of each class are pure and are produced by a single active source, as shown in Fig 3(a) and 3(b). After training, a speech mixture of two simultaneous active sources was given to the network and it classifies the time-frequency (TF) units as either belonging to the target (*s*_1_) or the interferer (*s*_s_), as shown in Fig 3(c). In the SONET speech separation model, two separate networks were trained. The first network i.e. the ILD-SONET is trained on the interaural level difference (ILD) spectrograms, and the second (the IPD-SONET) is trained on the interaural phase difference (IPD) spectrograms. The main motivation behind recycling SONET for the SD&D task is based on the fact that its training was done in anechoic conditions, where only the direct-path exists and only one source is active, so SONET [47] has already learned the direct-path spatial cues of that source during its training phase (when trained and tested for the speech separation task in [47]) and would not require any retraining or modification in architecture to recognize these cues in the presence of echoes (when used in Triple-0 model for the SD&D) as shown in Fig 3(d). In this digure, the TF units of audio mixture belonging to the noise and reverberations reaching the binaural setup act as zero-data for SONET which has never seen such conditions during its training phase. Another motivation for using SONET is that it is a TF masking speech separation algorithm, which estimates both the magnitude and the phase mask for the target source. It is found that the masking-based speech enhancement methods perform better than the mapping-based methods [28]. Also, the TF-masking in the complex domain (consisting of both the magnitude and phase) has been found beneficial when dealing with reverberation and noise [14].

### Triple-0 system overview

For any binaural source separation system with *Q* multiple simultaneous active sources, the mixture signals *x*_*k*_ collected at the *k*^*th*^ microphone of the binaural setup is given in [63] as

$$x_{k}(t)=s_{1}(t)+s_{2}(t)+\cdots +s_{q}(t)+\ldots +\;s_{Q}(t)+n_{k}(t)\;\;for\;k=\left\{1,\;2\right\}$$ (1)

where *s*_*q*_(*t*) = *S*_*q*_(*t*) * *h*_*kq*_ (*t*) is the signal collected at the microphones generated from target *S*_*q*_, *h*^*kq*^ represents the room impulse response between the active source *S*_*q*_ and the *k*^*th*^ microphone, ‘*’ is the convolution symbol, *n*_*k*_ is the diffuse noise source added in the mixture collected at the *k*^*th*^ microphone and *t* shows the discrete time index for the signal sampled at the frequency *f*_*s*_.

In our proposed setup, a single source *S* is placed in a reverberant noisy room, as shown in Fig 3(d). The early reverberation results in the creation of virtual sources that can be modeled with the image method [20]. In this method, the walls of the room are treated as acoustic mirrors. Each early reflection results in the creation of a virtual replica of the original source due to the law of reflection. This virtual source acts as if is located behind the wall symmetrically [16]. In the presence of multiple virtual sources (created due to the reverberations generated by the emission of the real source *S*), the mixture signal collected at the *k*^*th*^ microphone placed in a noisy room is given in [63] as

$$x_{k}(t)=s_{1}(t)+vs_{2}(t)+\cdots +vs_{p}(t)+\ldots +\;vs_{P}(t)+n_{k}(t)\;\;for\;k=\left\{1,\;2\right\}$$ (2)

where Eq (2) follows from Eq (1), with virtual sources replacing the real sources in Eq (1). The real source *s*_1_ in Eq (2) is given as *s*_1_(*t*) = S*h_*kS*_ (*t*), and *h*_*kS*_ represents the RIR between the source *S* and the *k*^*th*^ microphone. *VS*_1_,*VS*_2_,…..*VS*_*P*_ are virtual sources produced due to early echoes being specular [16]. Each virtual source in Eq (2) is given as *vs*_*p*_(*t*) = *VS*_*p*_(*t*)*h_*kp*_ (*t*) and *h*_*kp*_ represents the RIR between the virtual source *VS*_*p*_ and the *k*^*th*^ microphone. *P* is the total number of virtual sources created due to reverberations. The number of virtual sources *P* increases proportionally with the reverberation time (*RT*_*60*_) of the room.

As SONET [47] is a two-source anechoic separation system, trained and tested in noiseless conditions, the microphone signal *x*_*k*_ collected at the *k*^*th*^ microphone in case of the SONET speech separation system (shown in Fig 3(c)) is given as in Eq (3):

$$x_{k}(t)=s_{1}(t)+s_{2}(t)\;\;\;for\;k=\left\{1,\;2\right\}$$ (3)

In this case, *s*_1_ is the convolved speech signal produced by the target source *S*_*1*_, and *s*_2_ is the convolved speech signal produced by the interferer *S*_2_. SONET speech separation system has learnt the direct-path cues of *s*_1_ during its training phase, and later was able to identify these cues in the presence of unwanted cues produced by the interferer *s*_2_, when both sources are simultaneously active during the testing phase.

When the trained SONET is used for SD&D of a single active speech source *S* placed inside a noisy reverberant enclosure (shown in Fig 3(d)), the interferer signals are generated by all the virtual sources (*VS*_1_,*VS*_2_,…..*VS*_*P*_) and the diffuse noise source *n*_*k*_. All these virtual sources and the diffuse noise source are classified by SONET as interferers i.e. *s*_*2*_. Comparing Eqs (2) and (3), the interferer source *s*_2_ is given in Eq (4) as:

$$s_{2}(t)=\overset{P}{\underset{p=1}{\sum }}vs_{p}(t)+n_{k}(t)$$ (4)

Where *vs*_*p*_ is the convolved speech signal produced by the virtual source *VS*_*p*_.

In zero-shot testing for the SD&D task, SONET can identify the cues of target speech *s*_1_ (generated by the active source *S*), even in the presence of multiple virtual sources and noise. This is because of the excellent learning capabilities of neural networks and the unique training style of SONET separation system, where instead of training on the audio mixtures, training was done on the separate classes of target and interferer interaural cues, enabling the system to identify the target whether the interferer is a directional source (in case of speech separation (Eq (3))) or it is a set of many virtual sources (created due to reverberations) (in case of SD&D (Eq (4))).

**Fig 4:**
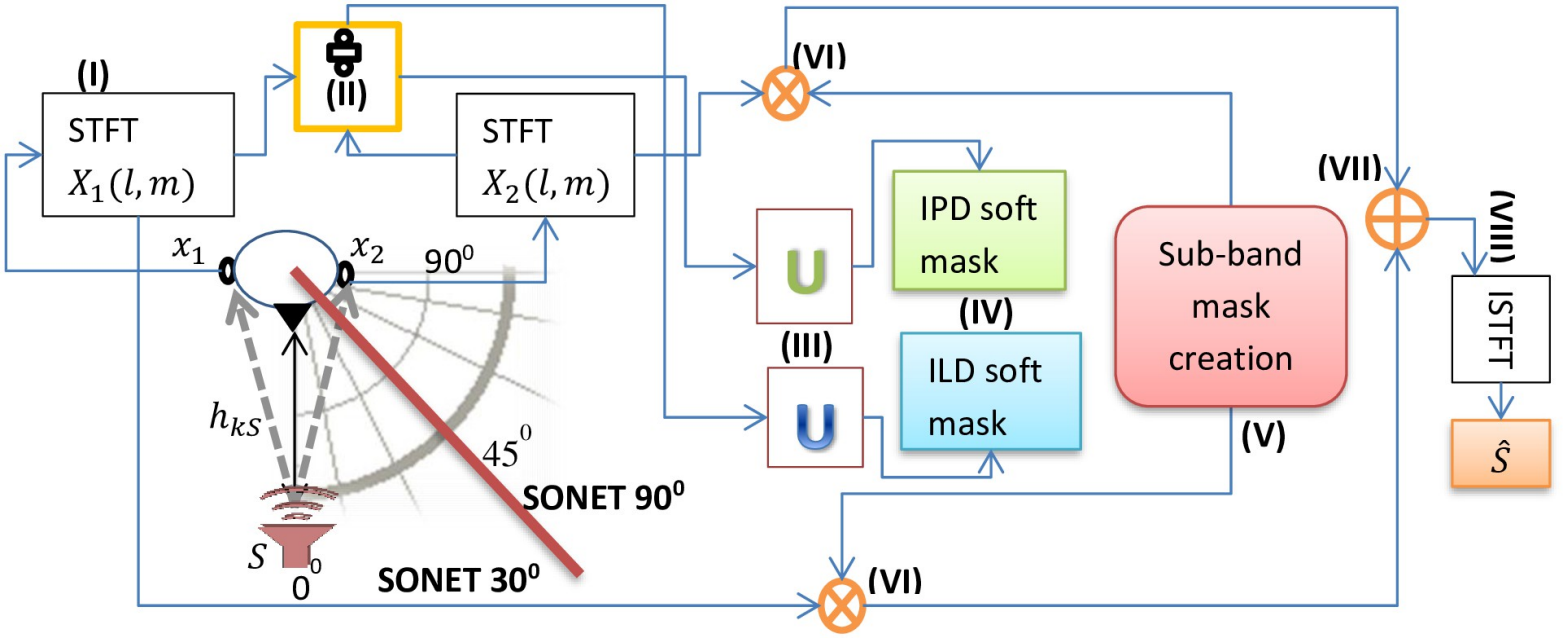
Equipment setup and processing steps (shown in boldfaced roman numbers enclosed in parenthesis) of the Triple-0 model. The source is currently making 0° with the binaural setup (manikin head). The source can be on the inner arc (radius 1m) or on the outer arc (radius 1.5m) arc around the head. The red line passing through 45° defines the SONET 30° and SONET 90° usage areas.

The equipment setup and processing steps of Triple-0 algorithm are shown in Fig 4.

The processing steps of Triple-0 are described in detail below.

**Step (I):** As SONET is designed to accept the interaural spectrograms at its input, so in the first step of signal processing, the noisy reverberant speech signal *x*_*k*_(*t*) is converted from the time domain to the time-frequency (TF) domain by taking its short-time Fourier transform (STFT) as given in Eq (5):
$$X_{k}(l,m)=Ƒ(w(t)x_{k}(t))$$ (5)
  where *l* represents the discrete frequency index, *m* represents the time frame index, Ƒ is the symbol representing the STFT operation, and *w*(*t*) represents the window function (hamming), mathematically given as *w*(*t*) = 0.54−0.46 cos (2*πt*/*N*), 0 ≤ *t* ≤ *N*, where *N* = *WL* − 1, and *WL* is the window length.
**Step (II):** In this step, the interaural spectrogram is generated by taking the ratio of the STFT of signals at both microphones, as given in Eq (6):
$$\frac{X_{1}(l,m)}{X_{2}(l,m)}=\alpha (l,m)e^{i\phi \left(l,m\right)}$$ (6)
  where *α*(*l*,*m*) is the ILD and *ϕ*(*l*,*m*) is the IPD at a TF point having discrete frequency index *l* and time frame index *m*. As ILD is the ratio of the energies at the left and right ear, it is expressed in decibels (dB) at each point of spectrogram by the formula given in [16, 57] as:
$$ILD(dB)=\;20log_{10}\alpha (l,m)$$ (7)
**Step (III):** In this step, the interaural (ILD and the IPD) spectrograms are given as inputs to the pretrained networks ILD-SONET and IPD-SONET respectively. These SONET networks are already trained on the direct-path cues of the sources placed at different azimuthal angles ranging from 0° to 90° (towards right) of the binaural setup as shown in Fig 3(a) and 3(b) for the source separation task in [47], so now they successfully recognize these cues even in the presence of echoes and noise as shown in Fig 3(d) and classify these cues as either belonging to the target class (*s*_1_) or the noise and reverberation class (*s*_2_). As stated in [47], the interaural cues are similar for the nearby locations, so the generalized solution of using only two pretrained SONET models i.e. SONET 30°, and SONET 90° for the sources placed at different angular separations will be used here too for SD&D. So if the target source *S* is placed at any angle within 0° to 45°, SONET 30° is used and if it is placed at any angle ranging from 45° to 90°, SONET 90° is activated. The domains of these networks are marked in Fig 4 by the red partition line coming out of the manikin head.
**Step (IV):** In this step, two soft masks are generated at the softmax layers of ILD and IPD-SONETs. The first one is for retrieving the direct-path signal coming from the target source and the second one is for extracting the reverberations and noise present in the incoming speech.
**Step (V):** A sub-band mask is constructed from the ILD and the IPD soft masks of the direct-path according to the strength of the cues in different bands [64]. The IPD cues are stronger than the ILD cues in the frequency band between 0 and 1.5 kHz, while both of them are weak in the region between 2 and 4 kHz and the ILD cues are very much stronger than IPD cues above 4 kHz due to the ‘head shadow’ effect. So, keeping the natural strength of spatial cues in mind, the sub-band mask is devised from the soft masks of the target produced by the ILD-SONET and the IPD-SONET, as shown in Fig 4 and given in Eq 8 as:
$$\begin{array}{ll} Sub-band\;mask & =[IPD\;mask\;\left(0-1.5\;kHz\right);\\ & Product\;of\;ILD\;and\;IPD\;masks\\ & \left(1.5-4\;kHz\right);ILD\;mask\;\left(4-8\;kHz\right)] \end{array}$$ (8)
  For converting the time-domain signal to time frequency domain, window length of 1024 samples with 25% overlap is used. For 1024-point STFT, Matlab generates 1024 (range: 1 ~1024) discrete frequency components for each time frame. Half of these components (from 1 to 512) represent positive frequencies and the other half is reserved for the negative frequencies (from 513 to 1024). The sub-band formation on the discrete frequency scale, corresponding to the analog bands given in Eq (8), is shown in Table 1.
**Step (VI), (VII) and (VIII):** In step (VI), the sub-band mask is multiplied elementwise to the STFT matrices of the reverberant and noisy speech *X*_1_(*l*,*m*) and *X*_2_(*l*,*m*) while in step VII, the outcomes of step (VI) are added together to retrieve the estimated STFT matrix of the dereverberated and denoised speech. This spectrogram is then converted back to the estimated time-domain target signal $\hat{S}$ by taking its inverse short-time Fourier transform (ISTFT) in step (VIII) and the estimated signal is then evaluated against the clean speech.

**Table 1:** Sub-band discrete frequency ranges.

Mask	Analog band	Discrete positive frequency range	Discrete negative frequency range
IPD mask	0–1.5 kHz	1–96	929–1024
Product of ILD and IPD masks	1.5–4 kHz	97–256	769–928
ILD mask	4–8 kHz	257–512	513–768

### Experimental parameters

This section includes the experimental setup, datasets, RIRs, objective quality and intelligibility metrics (used for the performance evaluation), and a brief description of the baseline algorithms, used for comparison of our proposed algorithm Triple-0.

### Experimental setup

The experimental setup is shown in Fig 3(d), where a single active source *S* is placed in a noisy echoic room. The reverberant conditions are simulated by convolving the source with the echoic binaural room impulse responses (BRIRs) taken from [45, 65]. The BRIRs of [45] are recorded by using the head and torso simulator (HATS) apparatus and that of [65] by Knowles electronic manikin for acoustic research (KEMAR) apparatus. According to these BRIRs, the distance between the two ears of the manikin (with miniature microphones inserted in them) replicates the human interaural spacing of 175 mm. The source-to-microphone distance is 1.5m in the case of [45] and it is 1m for [65]. SONET was trained only for the source-to-microphone distance of 1.5m when used for source separation [47]. It was not trained for 1m distance. As SONET was trained for the target positions varying in the range [0°:15°:90°] moving towards the right, it will be tested for dereverberation with the source *S* placed only at these positions.

### Dataset

Neural network models trained on one dataset must work seamlessly for other datasets without the need for retraining on these datasets [66]. So, apart from testing on the unseen data partitioned for model testing from the training dataset, the trend is to measure the network performance on completely disjoint datasets [50]. Examples of such network testing are: [66] for text classification, [52] for image classification, and [50] for audio source separation. Zero-shot learning not only allows scaling across unseen classes but also across unseen datasets [66]. As the pretrained model ‘SONET’ is trained on the clean audio files of the TIMIT dataset [67], it is required to test it not only for the unseen classes of the TIMIT dataset (i.e. noisy and reverberant TIMIT audio files) but also on a completely disjoint dataset (VCKT_DEMAND speech corpus [68]) to evaluate its performance across unseen classes and unseen datasets. These two datasets are among the most frequently used datasets for deep learning speech enhancement models [69]. Examples of the SD&D algorithms using TIMIT [67] are [70–72], while [73, 74] use the VCKT_DEMAND speech corpus [68, 75] uses both.

For zero-shot testing of Triple-0, ten speech samples are taken from the TIMIT dataset [67]. Each sample is of 2s duration, sampled at 16 KHz, and uttered by a different speaker. According to SD&D algorithms [12, 25, 29], the noisy samples for ZST of our proposed algorithm are created by adding white Gaussian noise at an SNR level of 20 dB. The total duration of the TIMIT test dataset is 20s. As SONET was also trained on TIMIT speech corpus, this dataset is called homo-corpus in the discussion ahead. To evaluate the proposed algorithm on hetero-corpus, Triple-0 will use the test dataset of the ‘28 speaker’ module of the VCKT_DEMAND speech corpus [68]. This dataset is composed of clean and noisy samples of two speakers (1 male and 1 female) sampled at 16 KHz. Five types of noises are selected from [76] and mixed with the clean speech samples taken from the voicebank corpus [77] at SNR values of 17.5, 12.5, 7.5, and 2.5dB. This results in 20 different conditions for the test set. The prepared noisy speech data is already available at the above-mentioned SNR values in the folder “noisy_testset_wav.zip” on the website [68]. The duration of the VCKT_DEMAND speech corpus testing set is around 34.5 minutes (= 2072s). All sound files from the clean and noisy speech are first concatenated to form a long audio signal and then cropped to 1036 samples of uniform 2s duration.

First the source signal (clean speech sample in case of noiseless conditions and noisy speech sample in case of noisy conditions) is normalized. Then it is convolved with the binaural room impulse responses (BRIRs) according to the source position inside the room to generate reverberant speech. The duration of the speech signal exceeds 2s after convolution but the signal is not cropped to keep the reverberations unimpaired. The signals collected at the binaural setup are then transformed into interaural spectrograms by using Eqs (5), (6), and (7), and given as input to the pretrained ILD and IPD-SONETs, and the dereverberated signal $\hat{S}$ is obtained by the procedure described in subsection ‘triple-0 system overview’.

This process is then repeated for all speech samples at each of the seven positions in the range [0°:15°: 90°] and the results are averaged within each room and then over all the rooms (A. B, C, D, and S). The baseline algorithms are also tested for the same speech samples. The duration of testing data for each algorithm is approximately 40 hours = 35 minutes (20sec (TIMIT) +2072sec (VCKT_DEMAND)) × 7 (positions) × 5 (rooms) × 2 conditions (noisy and noiseless).

### Binaural room impulse responses (BRIRs)

The BRIRs of [45, 65] are chosen for speech reverberation as these BRIRs have been used in many other state-of-the-art binaural SD&D algorithms e.g. [14, 57] and they are representative of the most of the real-world acoustic reverberant conditions. The dimensions and RT_60_s of the rooms where these BRIRs are recorded are given as: 1) Room S: *RT*_*60*_ = 560ms, dimensions = 5m × 9m × 3.5 m [65], 2) Room A: *RT*_*60*_ = 320ms, dimensions = 6.6m × 5.7m × 2.3m, 3) Room B: *RT*_*60*_ = 470ms, dimensions = 4.6m × 4.6m ×2.6m, Room C: *RT*_*60*_ = 680ms, dimensions = 18.8m × 23.5m × 4.6 m, and Room D: *RT*_*60*_ = 890ms, dimensions = 8.7m × 8m × 4.25 m [45]. The details can be found in [45, 65].

For room S, the source-to-microphone setup spacing is 1m, whereas for other rooms it is 1.5m, shown respectively by the inner and outer arcs around manikin’s head in Fig 4. *RT*_*60*_ usually increases as the volume of the room increases [78]. But this is not the case in room C. One of the reasons for this principle violation is that apart from the volume, the *RT*_*60*_ also changes with the shape of the room, the absorption coefficient of ceiling, floor, and walls, the audience, and the kind of seating inside the room [79]. Room C is a large lecture theatre having a low ceiling around the lectern and an abundance of soft seating, resulting in a relatively small *RT*_*60*_ as opposed to its volume [45].

### Objective evaluation metrics

Signal-to-distortion ratio (SDR), perceptual evaluation of speech quality (PESQ), speech-to-reverberation modulation energy ratio (SRMR), short-term objective intelligibility (STOI), and cepstral distance (CD) are the objective evaluation metrics used for the comparison of our proposed algorithm with other baseline algorithms. For all metrics (except CD), higher value means better. SRMR is a non-intrusive metric, so no reference signal is required for its estimation [80], whereas the rest of the metrics are intrusive metrics and thus require the clean speech sample as a reference for the performance evaluation [80]. Among these metrics, PESQ and STOI are known to correlate well with the human perception of quality and intelligibility [81]. SRMR metric is commonly used to evaluate speech dereverberation algorithms and reflect the quality and intelligibility of the reverberant speech [57]. SDR shows the estimated speech quality by comparing the estimated signal energy with all kinds of distortions [81]. CD measures the similarity between short-time spectra of the estimated and clean speech [81].

### Baseline algorithms

For comparing the performance of our proposed algorithm, two baseline algorithms are selected 1) weighted prediction error (WPE) [82] and 2) a U-Net-based pretrained network dereverb (PND) model [29]. WPE belongs to the group of ‘classical algorithms’ and PND is a deep learning-based algorithm. These two algorithms are chosen for three important reasons. Firstly, they do not require any kind of training before testing them on our data. Secondly, the WPE algorithm is the best and the most widely used signal processing-based dereverberation algorithm [12] and thirdly the PND model [29] is very much similar to our proposed model as it is also an audio-only SD&D model based on U-Net. The main difference between the PND model [29] and our proposed model is that [29] is designed, trained, and tested specifically for the SD&D task, whereas the pretrained model SONET that is being used in our proposed SD&D algorithm is designed especially for the anechoic speech separation of only two sources in the noiseless condition.

A brief overview of these algorithms is given below.

The WPE [82] is a dereverberation algorithm that can blindly shorten the RIR by incorporating the linear prediction filters. This method offers a generalized dereverberation solution which is computationally efficient and tested to be effective under a variety of reverberant conditions. Due to introducing minimum distortion to speech, WPE is mostly used in automatic speech recognition (ASR) applications [12]. Among different approaches, investigated in the literature, WPE has exhibited promising results for dereverberation. However, its performance decays rapidly in the presence of even a small amount of additive noise [83]. The PND algorithm [29]; an SD&D algorithm, uses U-Net and needs log-spectrograms as input. The network is only trained on the magnitude spectrograms of the training dataset and utilizes the noisy phase for speech reconstruction during the testing phase. The network exhibits better performance when asymmetric filters (larger for the frequency domain, and smaller for the time domain) are used in the convolutional layers of U-Net, as these filters exhibit better performance for audio signals than the conventional symmetric filters, used in the image processing CNNs [29].

## Results

### Case 1: Homo-corpus testing

The average results of different algorithms under both noisy and noiseless conditions in the five rooms (A. B, C, D, and S) for the TIMIT speech corpus are shown in Table 2.

**Table 2:** Comparison of different algorithms on TIMIT dataset.

**Noiseless conditions**					
**Algorithm**	**SDR (dB)**	**STOI (%)**	**SRMR (dB)**	**PESQ**	**CD**
[82]	2.2	82	4.1	2.2	5.3
[29]	0.1	83	4.2	1.8	**4.7**
**Proposed**	**3.2**	**86**	**5.5**	**2.5**	7.6
**Noisy conditions**					
[82]	6.9	75	4.7	**1.4**	6.4
[29]	5	**78**	5.0	**1.4**	**5.9**
**Proposed**	**7.8**	**78**	**5.1**	**1.4**	8.0

The best results for each metric are boldfaced. As clear from Table 2, except for CD, Triple-0 outperforms the baseline algorithms on all other metrics under noiseless conditions. Under noisy conditions, the SDR and SRMR are highest for our proposed model, while its PESQ is equal to the other baseline systems and its STOI is equal to the PND model [29]. The performance of Triple-0 is comparable to [29] even though it was never trained for noisy reverberant conditions, while [29] is trained for such conditions. Under these conditions, again the CD of our proposed algorithm is the largest of all systems, although its difference with other methods drops significantly as compared to the noiseless conditions.

### Case 2: Hetero-corpus testing

The average results of different algorithms under both noisy and noiseless conditions in the five rooms (A. B, C, D, and S) for the VCKT_DEMAND speech corpus are shown in Table 3.

**Table 3:** Comparison of different algorithms on VCKT_DEMAND dataset.

**Noiseless conditions**					
**Algorithm**	**SDR (dB)**	**STOI (%)**	**SRMR (dB)**	**PESQ**	**CD**
[82]	7.5	73	4.8	1.5	5.2
[29]	5.3	**80**	**5.1**	**1.6**	**4.1**
**Proposed**	**8.2**	78	**5.1**	1.4	6.6
**Noisy conditions**					
[82]	5.9	67	**4.5**	1.3	6.0
[29]	3.5	**75**	3.8	**1.4**	**5.5**
**Proposed**	**6.1**	73	3.5	1.3	7.6

Again the SDR of our proposed algorithm is the highest among all the baseline systems while its STOI, PESQ, and SRMR lag slightly behind the dedicated deep learning SD&D algorithm PND [29]. The reason may be the large amount of reverberant data used for the training of the PND model, trained on data of REVERB challenge [84], synthetic data using simulated RIRs of 24 rooms, and at various source-to-microphone distances, resulting in better generalization of the model under unseen conditions than our proposed model, which was trained for only anechoic conditions and on a single value of source-microphone spacing. The overall performance of both deep learning algorithms ([29] and Triple-0) is better than the signal processing-based algorithm [82] at all metrics except CD where Triple-0 even lags WPE [82]. Triple-0 is unsuitable for machine listening applications e.g. ASR and automatic speaker verification (ASV) due to its high value of CD, which indicates high spectral distortion [85]. However, Triple-0 can be used for assisted listening applications required for people with hearing problems in noisy echoic conditions and for normal-hearing and non-native listeners in adverse acoustic conditions, requiring speech enhancement.

## Conclusion

When compared to conventional signal processing and machine learning techniques, speech enhancement by deep learning has improved the performance but at the cost of high computational resources, large training datasets, and lengthy training durations. The use of pretrained networks (transfer learning) needs fine-tuning on the new task. Still, the system performance degrades for the unseen conditions. ZST is required to evaluate the system’s performance for the unseen classes. Our proposed algorithm Triple-0 is the ZST of the frozen U-Net-based anechoic source separation model SONET [47] for the SD&D task. SONET was trained only in the anechoic noiseless conditions but using it for Triple-0 has shown that it performs better than the most promising signal processing dereverberation algorithm WPE and is almost equivalent to a dedicated deep learning-based algorithm PND under a variety of reverberant and noisy conditions, and distances between the source and the binaural set-up and also on the unseen dataset without requiring any fine-tuning for the SD&D task. Due to deep learning, the anechoic pretrained model SONET is able to identify the direct-path cues generated by the target source even in the presence of echoes and noise. However, due to large CD as compared to other methods, Triple-0 is not appropriate for machine listening applications. Also, as the model is based on spatial cues, ZST for sources located far from the learned cues would result in performance degradation. Similarly, Triple-0 and the PND algorithm [29] require the audio conversion to spectrogram that is not required for WPE [82], but this drawback is compensated by the enhanced performance provided by these systems compared to WPE. In this work Triple-0 is tested for diffuse noise sources. However, in future, the proposed model is also required to be tested for denoising the interference produced by multiple directional noise sources placed in reverberant conditions. Many pretrained audio processing models exist and their use for tasks other than those for which they are designed may prove beneficial not only in improving performance but also in saving time and computational resources.
